# LIM Mineralization Protein-1 Enhances the Committed Differentiation of Dental Pulp Stem Cells through the ERK1/2 and p38 MAPK Pathways and BMP Signaling

**DOI:** 10.7150/ijms.70411

**Published:** 2022-07-18

**Authors:** Rui Mu, Bo Chen, Bo Bi, Hongchuan Yu, Juan Liu, Junxia Li, Maodian He, Liang Rong, Bingyao Liu, Ke Liu, Lei Zhu, Xiaolei Shi, Yi Shuai, Lei Jin

**Affiliations:** 1 Department of Stomatology, Jinling Hospital, Medical School of Nanjing University, School of Stomatology of Southern Medical University, Clinical Medical School of Nanjing Medical University, Nanjing 210002, China.; 2 Stomatology Center, Peking University Shenzhen Hospital, Shenzhen Peking University-The Hong Kong University of Science and Technology Medical Center, Guangdong province, Shenzhen 518036, China.

**Keywords:** Dental pulp stem cells, Differentiation, LIM mineralization protein-1, Mitogen-activated protein kinase pathway, Tissue regeneration

## Abstract

Tissue regeneration is the preferred treatment for dentin and bone tissue defects. Dental pulp stem cells (DPSCs) have been extensively studied for their use in tissue regeneration, including the regeneration of dentin and bone tissue. LIM mineralization protein-1 (LMP-1) is an intracellular non-secretory protein that plays a positive regulatory role in the mineralization process. In this study, an LMP-1-induced DPSCs model was used to explore the effect of LMP-1 on the proliferation and odonto/osteogenic differentiation of DPSCs, as well as the underlying mechanisms. As indicated by the cell counting kit-8 assay, the results showed that LMP-1 did not affect the proliferation of DPSCs. Overexpression of LMP-1 significantly promoted the committed differentiation of DPSCs and vice versa, as shown by alkaline phosphatase activity assay, alizarin red staining, western blot assay, quantitative real-time polymerase chain reaction assay, and *in vivo* mineralized tissue formation assay. Furthermore, inhibiting the activation of the extracellular signal-regulated kinase 1/2 (ERK1/2), p38 mitogen-activated protein kinase (MAPK), and c-Jun N-terminal kinase (JNK) pathways using specific pathway inhibitors showed that the ERK1/2 and p38 MAPK pathways attenuated the differentiation of DPSCs. Besides, the expression of BMP signaling pathway components were also determined, which suggested that LMP-1 could activate BMP-2/Smad1/5 signaling pathway. Our results not only indicated the underlying mechanism of LMP-1 treated DPSCs but also provided valuable insight into therapeutic strategies in regenerative medicine.

## Introduction

Disease and trauma have resulted in a high incidence of dentin or alveolar bone defects, which severely affect the quality of life of patients. Constructing a dentin/bone tissue regeneration therapy system has become a hot focus in the field of tissue engineering and regenerative medicine. The tissue engineering technology, which utilizes the multi-differentiation potential capacity of stem cells, is expected to be used to repair damaged tissues or organs.

Mesenchymal stem cells (MSCs) have the potential for self-renewal and multi-directional differentiation 1; thus, MSCs-based cellular therapies have been considered for the repair of damaged tissue 2. MSCs can be isolated from a wide range of sources, such as bone marrow, peripheral blood, dental tissue, and the placenta 3. Compared with MSCs from other sources, dental stem cells can be obtained more conveniently and are considered to be a promising candidate for tissue engineering and regenerative medicine.

Dental pulp stem cells (DPSCs), a type of odontogenic mesenchymal stem cell that originates from the transitional nerve crucible cells, are isolated from dental pulp tissue and have been demonstrated to have the ability to differentiate into odontoblasts, osteoblasts, chondroblasts, neuroblasts, fibroblasts, vascular cells, endothelial cells, pericyte-like cells, smooth muscle-like cells, and inner ear hair cells 4-11. In the appropriate microenvironment, DPSCs can be used as seed cells to differentiate into odontoblasts and regenerate the pulp-dentin complex, which may assist in tooth reparation, root regeneration and even functional tooth regeneration using tissue engineering methods 12. DPSCs have shown specific surface markers and matrix proteins that are associated with mineralization formation, which are similar to the well-known bone marrow mesenchymal stem cells (BMMSCs). However, differentiation of these stem cells usually relies on the participation of growth factors.

LIM mineralization protein 1 (LMP-1) is a member of the LIM domain protein family that contains a full-length and conserved PDZ domain and LIM domains, and a non-conserved sequence. LMP-1 also has exhibited the potential for osteogenesis 13. It is an intracellular osteogenic factor associated with bone development and promotion of the expression of various osteogenic growth factors, such as bone morphogenetic proteins (BMPs) and transforming growth factor β (TGF-β), during the differentiation of osteoblasts and the formation of bone mineralization 13-16. Some studies have also shown that LMP-1 could promote osteogenesis through activation of the extracellular signal-regulated kinase (ERK) signaling pathway in embryonic stem cells and osteogenesis cells 17, 18. Dentin and bone tissue are highly similar in terms of developmental origin, mechanism of formation, and tissue composition 19, 20. Many growth factors are not only involved in the formation and reconstruction of bone tissue but also play a vital role in the construction and mineralization of dentin 21, 22. Researchers have suggested that LMP-1 is mainly expressed in predentin, odontoblasts, and vascular endothelial cells of healthy teeth. However, LMP-1 was also determined to be expressed in unmineralized reparative dentin, odontoblast-like cells, and dental pulp cells in dental caries and pulpitis teeth 23. Therefore, these results indicated that LMP-1 was an indispensable factor in the regulation of odontogenic tissue development and regeneration.

Our previous studies focused on MSCs regulation and its application in odonto/osteogenic tissue formation improvement. Compared with BMMSCs, DPSCs showed similar surface markers and matrix proteins associated with mineralization formation 24. Till date, LMP-1 has been verified to enhance the osteogenesis of BMMSCs. However, the exact effect of LMP-1 on DPSCs and the underlying mechanisms involved are still largely unknown. It would be of great interest to investigate the effect of LMP-1 on the committed differentiation of DPSCs and its regulatory mechanism, which may provide an effective treatment strategy for the regeneration of dentin and bone tissue.

## Materials and methods

### Cell isolation and culture

Human third molars without caries and inflammation were collected from healthy donors (18-25 years old) at Nanjing Jinling Hospital of Southern Medical University. The study procedures were approved by the Medical Ethics Committee of Nanjing Jinling Hospital (approval number: 2020DZGZRZX-077) and performed with the informed consent of the patients. The pulp tissue was carefully isolated and digested in a solution of 3 mg/mL type I collagenase and 4 mg/mL dispase (Sigma-Aldrich, St. Louis, USA) for 1 h at 37 °C. The dissolved tissue was incubated in the alpha minimum essential medium (α-MEM; Gibco, USA) supplemented with 10% fetal bovine serum (FBS; Gibco, Life Technologies, USA), 10,000 units/mL penicillin, and 10,000 μg/mL streptomycin (penicillin-streptomycin solution; HyClone, USA) at 37 °C in 5% CO_2_. The signaling pathway-specific inhibitors used were the ERK1/2 pathway inhibitor (U0126, Selleck, USA), p38 MAPK pathway inhibitor (SB203580, Selleck, USA), and Jun N-terminal kinase (JNK) pathway inhibitor (SP600125, Selleck, USA). The culture medium was replaced every 2 days. Third-passage cells were harvested for the subsequent experiments.

### Flow cytometry

DPSCs were collected at a density of 1×10^5^ cells/tube using trypsin. The cells were rinsed twice with phosphate-buffered saline (PBS) with 3% FBS. After adding primary antibodies, which included STRO-1 (MA5-28636, eBioscience, CA, USA), CD29 (11-0299, eBioscience, CA, USA), CD34 (12-0349, eBioscience, CA, USA), CD45 (11-9459, eBioscience, CA, USA), CD90 (11-0909, eBioscience, CA, USA), CD105 (12-1057, eBioscience, CA, USA), and CD146 (11-1469, eBioscience, CA, USA), into each solution, the samples were incubated for 1 h at room temperature in the dark. Cell surface markers were identified by flow cytometry (Biolegend, USA).

### Lentivirus infection

The lentivirus-LMP-1 (LV-LMP-1) and lentivirus-control (LV-vector) of lentiviral vector-mediated overexpression were obtained from Jikai Gene Chemical Technology Co., Ltd. (Shanghai, China) (catalog number: 16046-1). The lentivirus-shLMP-1 (LV-shLMP-1) and lentivirus-scramble control (LV-scramble) of lentiviral vector-mediated low-expression were also obtained from the same company (catalog number: 75900-1). After DPSCs were transfected by lentiviruses in α-MEM with 5 μg/mL polybrene for 24 h, the medium was changed using a fresh complete medium according to the manufacturer's instructions.

### Cell proliferation assay

The proliferation of transfected DPSCs was assessed using the cell counting kit-8 assay (CCK-8) (Dojindo, Japan). DPSCs were plated onto 96-well plates with 5×10^3^ cells/well and incubated for 24 h at 37 °C in 5% CO_2._ The α-MEM medium with 10% FBS was replaced after cell attachment. After incubation days 1, 3, 5, 7, and 9, cells were treated with CCK-8 reagents at 37 °C for 2 h according to the manufacturer's instructions. Optical density (OD) was detected at 450 nm using a microplate reader (Infinite f50, Tecan, Switzerland).

### Alkaline phosphatase (ALP) activity

Transfected DPSCs were cultured for 7 days and replaced with fresh medium every other day. The cells were then lysed in RIPA buffer (Beyotime, China), and the supernatant was collected for the addition of substrates and *p*-nitrophenol according to the manufacturer's instructions (Beyotime, China). After 30 min of incubation at room temperature, ALP activity was determined at a wavelength of 405 nm.

### Alizarin red staining and quantification

Alizarin red staining was performed to evaluate the mineralization ability of cells 25. Transfected DPSCs were seeded in six-well plates at a density of 5 × 10^5^ cells/well and cultured with mineralization inducing medium containing α-MEM, 5% FBS, penicillin-streptomycin solution, 10 nmol/L dexamethasone (Sigma, USA), 50 mg/L ascorbic acid (Sigma, USA), and 10 mmol/L β-glycerophosphate (Sigma, USA) for 14 days and then processed for alizarin red staining (Leagene, Beijing, China). For quantitative analysis, the mineralized nodules were dissolved in 10% cetylpyridinium chloride (CPC; Amresco, USA) in phosphate buffer for 30 min and detected by spectrophotometric absorbance at 562 nm.

### Quantitative real-time polymerase chain reaction (qRT-PCR)

Total RNA was extracted from DPSCs using TRIzol reagent (Ambion, Carlsbad, USA). The mRNA was reverse-transcribed into cDNA using the HiScript Q RT SuperMix kit (Vazyme, China). qRT-PCR was performed to detect LMP-1, dentin sialophosphoprotein (DSPP), dentin matrix protein 1 (DMP-1), osteocalcin (OCN), and runt-related transcription factor 2 (RUNX2), which were then quantitated with FastStart Universal SYBR-Green Master (Roche, Indianapolis, USA) and a BioRad CFX96 real-time PCR detection system (Biorad, Hercules, USA). Glyceraldehyde-3-phosphate dehydrogenase (GAPDH), a housekeeping gene, was employed for endogenous normalization, and the relative gene expression values were calculated using the 2^-ΔΔCt^ method. The primers used for these genes were as follows: LMP-1 (forward 5'-CGGGATCCATGGATTCCTTCAAGGTAGTG-3', reverse 5'-CGCTCGAGTCACACATGAGAGAAGGCATG-3'), DSPP (forward 5'-TTTGGGCAGTAGCATGGGC-3', reverse 5'-CCATCTTGGGTATTCTCTTGCCT-3'), DMP-1 (forward 5'-AGGAAGTCTCGACTCTCAGAG-3', reverse 5'-TGGAGTTGCTGTTTTCTGTAGAG-3'), OCN (forward 5'-CCCAGGCGCTACCTGTATCAA-3', reverse 5'-GGTCAGCCAACTCGTCACAGTC-3'), RUNX2 (forward 5'-GAATGCCTCTGCTGTTATG-3', reverse 5'-ACTCTTGCCTCGTCCACT-3'), GAPDH (forward 5'-GGAGCGAGATCCCTCCAAAAT-3', reverse 5'-GGCTGTTGTCATACTTCTCATGG-3').

### Western blot

Cells were lysed in RIPA buffer, and total protein concentrations were quantified using a bicinchoninic acid assay kit (Pierce, Rockford, USA). The same amounts of proteins were separated using sodium dodecyl sulfate-polyacrylamide gel electrophoresis and transferred through electroblotting to polyvinylidene fluoride membranes (Roche, Indianapolis, USA). The membrane was blocked with 5% fat-free milk in TBST for 2 h at room temperature and incubated overnight with primary antibodies, which included LMP-1 (10221-1-AP, Proteintech, IL, USA), BMP-2 (66383-1-Ig, Proteintech, IL, USA), BMPRⅠA (12702-1-AP, Proteintech, IL, USA), BMPRⅡ (14376-1-AP, Proteintech, IL, USA), DSPP (sc-73632, Santa Cruz, TX, USA), DMP-1 (sc-73633, Santa Cruz, TX, USA), OCN (sc-390877, Santa Cruz, TX, USA), RUNX2 (sc-390351, Santa Cruz, TX, USA), ERK1/2 (#4695, Cell Signaling Technology, MA, USA), p-ERK1/2 (#4376, Cell Signaling Technology, MA, USA), p38 (#8690, Cell Signaling Technology, MA, USA), p-p38 (#4511, Cell Signaling Technology, MA, USA), JNK (#9252, Cell Signaling Technology, MA, USA), p-JNK (#4668, Cell Signaling Technology, MA, USA), p-Smad1/5 (#9516, Cell Signaling Technology, MA, USA), Smad5 (#12534, Cell Signaling Technology, MA, USA) and GAPDH (60004-1-Ig, Proteintech, IL, USA).

### Odonto/osteogenic tissue formation *in vivo*

DPSCs were transfected with either the LMP-1 overexpression group (LV-LMP-1 and LV-vector) or with the low-expression group (LV-shLMP-1 and LV-scramble) and were subsequently incubated in an osteogenic medium for 1 week and harvested for the *in vivo* study. A gelatin scaffold (Kuaikang, Guangzhou, China) was chosen as a potential scaffold for tissue regeneration. Transfected cells were loaded on the gelatin scaffold at a density of 5 × 10^5^ cells/scaffold for 24 h at 37 °C and then implanted into the subcutaneous space of BALB/c homozygous nude mice (6 weeks old, five mice in each group) for 8 weeks to be used in subsequent experiments. The study procedures were approved by the Laboratory Animal Ethics Committee of Nanjing Jinling Hospital (Approval number: 2020JLHGKJDWLS-45).

### Histological and immunohistological analysis

The implants were collected and fixed in 4% paraformaldehyde solution (Biosharp, Hefei, China) and then decalcified, dehydrated, and embedded in paraffin. Tissue sections were stained with hematoxylin-eosin (H&E; Beyotime, Shanghai, China), and Masson's trichrome (Solarbio Life Science, Beijing, China). In the Masson's trichrome staining, the collagen and non-collagen components were distinguished by gray adjustment, the percentage of collagen tissue in each visual field was calculated (collagen area/measured visual field area), and the mean value was taken as collagen volume fraction (CVF). Immunohistochemical analysis of DSPP and OCN protein expression was also performed using a Histostain-Plus IHC Kit (Jiancheng Bioengineering Institute, China) according to the manufacturer's instructions. For immunohistological analysis, the slices were probed using the following primary antibodies: mouse anti-human monoclonal DSPP antibody (sc-73632, Santa Cruz, TX, USA) and rabbit anti-human monoclonal OCN antibody (23418-1-AP, Proteintech, IL, USA), and then incubated with the secondary anti-mouse IgG and anti-rabbit IgG, respectively. And the mean optical density (MOD) of protein expression was measured (integrate optical density/area) by using Image-pro Plus.

### Statistical analysis

Statistical analysis was performed via the SPSS 23.0 software (SPSS, IL, USA) and presented as the mean ± SD from three independent experiments. For measurement variables with a normal distribution and homogeneity of variance, a Student's t-test was used to determine the statistical significance of the differences between the two groups. A *P*-value of <0.05 was considered statistically significant.

## Results

### Characterization and transfection of DPSCs

**Figure 1:**
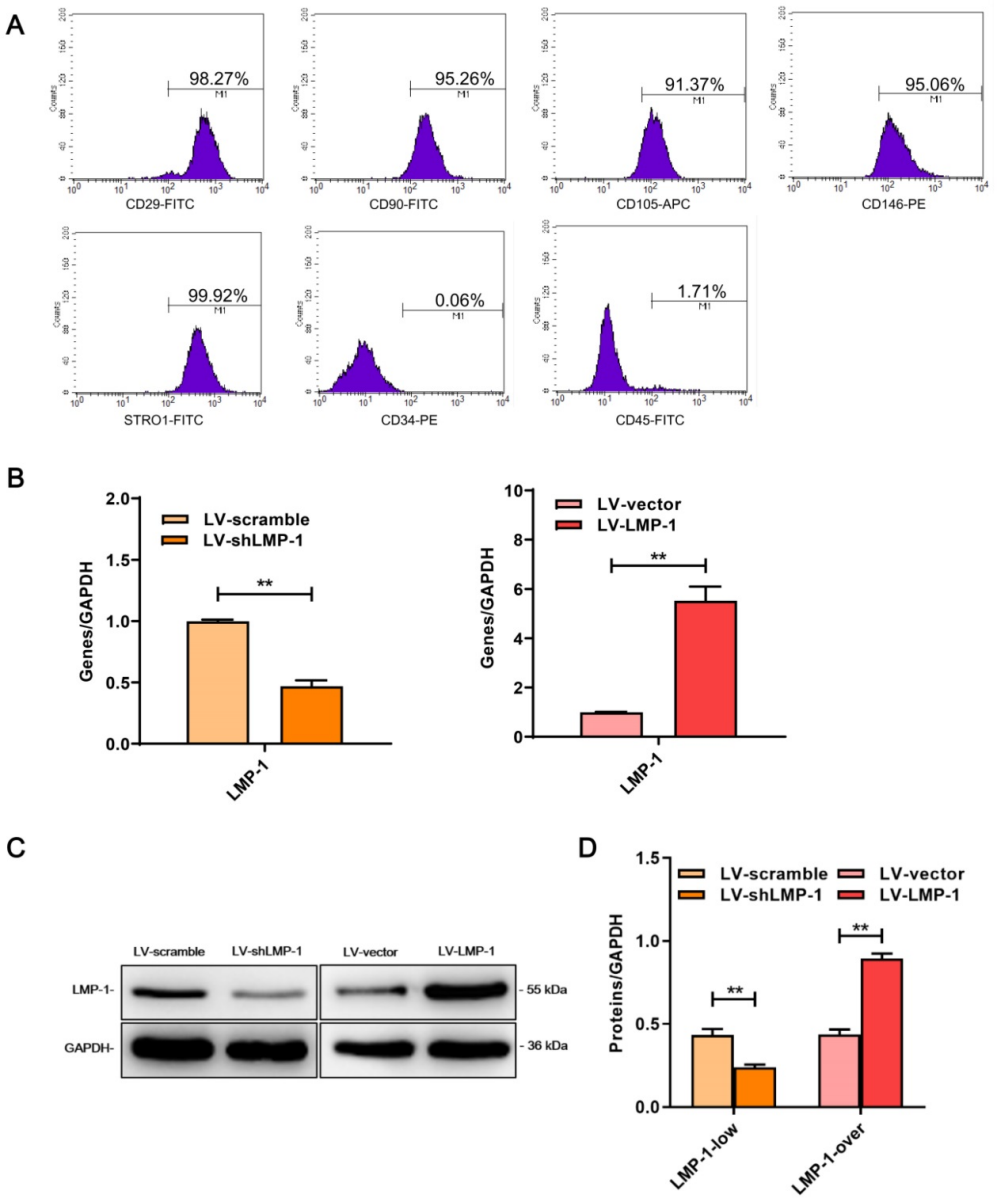
** Characterization of DPSCs and LMP-1 expression in transfected DPSCs. (A)** Flow cytometry demonstrated that the expression of CD29, CD90, CD105, CD146, and STRO-1 were positive in DPSCS, which were negative against CD34 and CD45. **(B)** Relative mRNA expression of LMP-1 in LV-scramble group, LV-shLMP-1 group, LV-vector group and LV-LMP-1 group after 3 days transfected in DPSCs, respectively (*P < 0.05, **P < 0.01). **(C)** Western blot analysis of LMP-1 protein expression in LV-scramble group, LV-shLMP-1 group, LV-vector group, and LV-LMP-1 group after 3 days transfected in DPSCs, respectively. **(D)** Grayscale analysis of (C) by ImageJ software (**P < 0.01).

The MSCs markers (STRO-1, CD29, CD90, CD105, and CD146) had a high expression level, whereas the hematopoietic markers (CD34 and CD45) had a low expression in DPSCs **(Figure 1A)**, which indicated that DPSCs that were extracted had a MSCs phenotype.

To detect the transfection efficiency, the expression level of LMP-1 in DPSCs was measured using qRT-PCR and western blot after 3 days of transfection. The results showed that LV-LMP-1 considerably increased the expression of LMP-1 as compared with that in the LV-vector of the overexpression group, whereas the expression of LMP-1 significantly decreased in LV-shLMP-1 in comparison with that in the LV-scramble of the low-expression group **(Figure 1B-D)**. Thus, the lentiviral vector could successfully transfect DPSCs and regulate the expression level of LMP-1, which laid a foundation for the subsequent experiments in the present study.

### LMP-1 did not affect the proliferation of DPSCs

**Figure 2:**
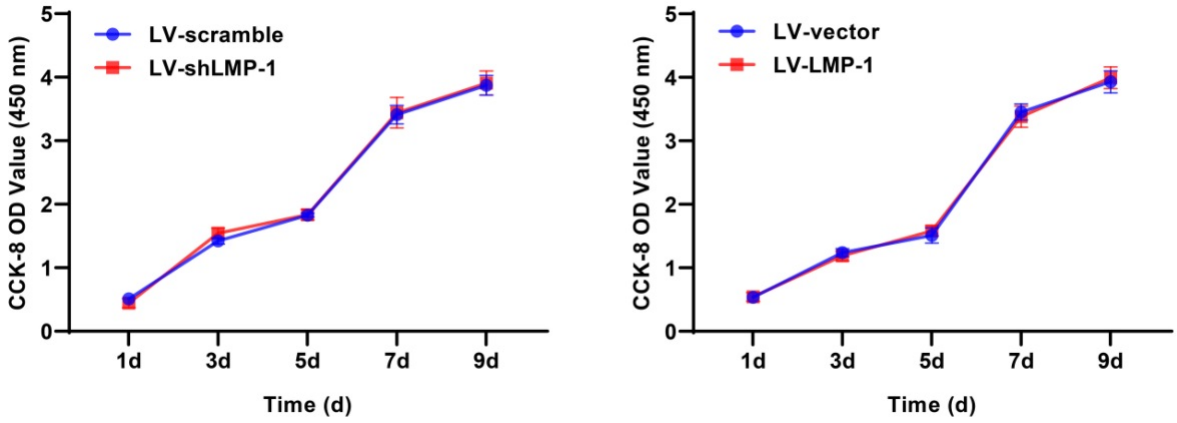
Effects of LMP-1 on the proliferation of DPSCs. CCK-8 assay showed no effect when LMP-1 low-expression or overexpression at day 1, 3, 5, 7, and 9, respectively.

A CCK-8 assay was performed to investigate the ability of DPSCs to proliferate after lentivirus transfection. The results did not show a significant difference in the proliferative rate between LV-LMP-1 and LV-vector; the LV-shLMP-1 and LV-scramble also did not have any significant difference in proliferative capacity **(Figure 2)**. The results indicated that LMP-1 did not affect the proliferation of DPSCs.

### LMP-1 promoted the odonto/osteogenesis of DPSCs

**Figure 3:**
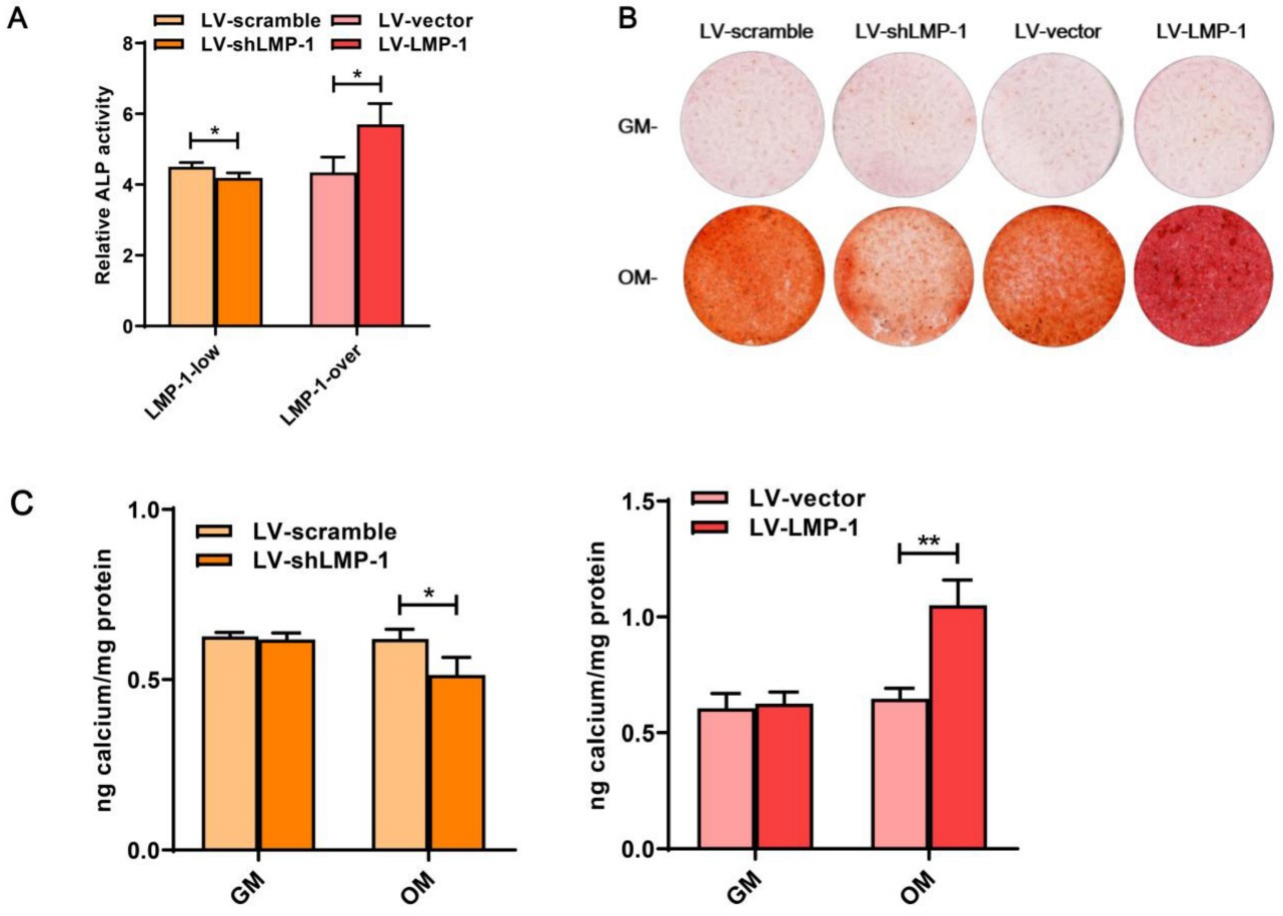
** Effects of LMP-1 on ALP activity and mineralized formation capacity of DPSCs. (A)** ALP activity of LMP-1 transfected DPSCs in LV-scramble group, LV-shLMP-1 group, LV-vector group, and LV-LMP-1 group after 7 days cultured in osteogenic medium (*P < 0.05). **(B)** The formation of mineralized nodules measured by alizarin red staining in four groups after transfected DPSCs cultured in growth medium (GM) or osteogenic medium (OM) for 14 days. **(C)** Quantification of alizarin red staining in four groups by CPC assay (*P < 0.05, **P < 0.01).

**Figure 4:**
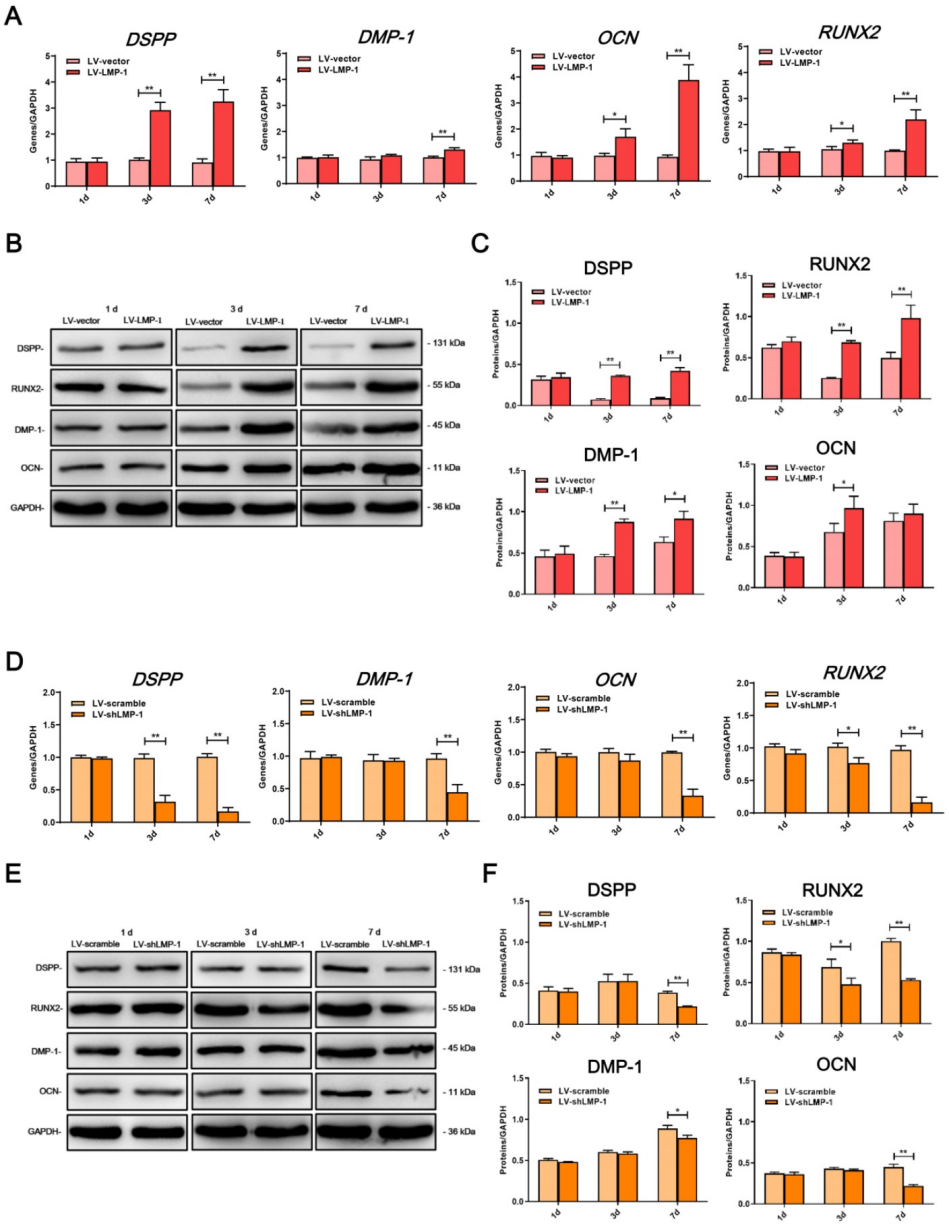
** Effects of LMP-1 on the odonto/osteogenic differentiation of DPSCs. (A)** Relative mRNA expression of DPSS, DMP-1, OCN and RUNX2 in LV-vector group and LV-LMP-1 group at day 1, 3 and 7, respectively (*P < 0.05, **P < 0.01).** (B, C)** Western blot analysis of LMP-1 protein expression in LV-vector group and LV-LMP-1 group at day 1, 3, and 7, respectively (*P < 0.05, **P < 0.01). **(D)** Relative mRNA expression of DPSS, DMP-1, OCN and RUNX2 in LV-scramble group and LV-shLMP-1 group at day 1, 3 and 7, respectively (*P < 0.05, **P < 0.01). **(E, F)** Western blot analysis of LMP-1 protein expression in LV-scramble group and LV-shLMP-1 group at day 1, 3, and 7, respectively (*P < 0.05, **P < 0.01).

In comparison with the LV-vector, the ALP activity was upregulated in the LV-LMP-1 transfected cells after 7 days of incubation **(Figure 3A)**. Meanwhile, after 14 days of osteogenic induction, LV-LMP-1 cells generated more mineralized nodules in comparison with the cells in the LV-vector **(Figure 3B)**. A CPC assay also indicated that the calcium concentration in the LV-LMP-1 group was much higher than that in the LV-vector group **(Figure 3C)**. In contrast, after the suppression of LMP-1 in DPSCs, the ALP activity was considerably lower than that in the LV-scramble **(Figure 3A)**. The mineralized nodules were markedly decreased in the LV-shLMP-1 group in comparison with those in the LV-scramble group **(Figure 3B-C)**. Afterward, the mRNA expressions of DSPP, DMP-1, OCN, and RUNX2 in the transfected cells under osteogenic conditions were measured using qRT-PCR, which indicated that mRNA expressions of these odonto/osteoblastic genes were upregulated at day 3 or day 7 after overexpressing LMP-1 in DPSCs **(Figure 4A)**. The protein levels of DSPP, RUNX2, DMP-1, and OCN also increased at day 3 or day 7 **(Figure 4B-C)**. In contrast, the mRNA expressions of DSPP, DMP-1, OCN, and RUNX2 were downregulated at day 3 or day 7 after the inhibition of LMP-1 in DPSCs **(Figure 4D)**, whereas the protein levels of DSPP, RUNX2, DMP-1, and OCN were also significantly decreased at day 3 or day 7 **(Figure 4E-F)**. These results suggested that the overexpression of LMP-1 promoted odonto/osteogenic differentiation of DPSCs by regulating the expression levels of corresponding genes and proteins.

### Histological and immunohistochemical analysis

**Figure 5:**
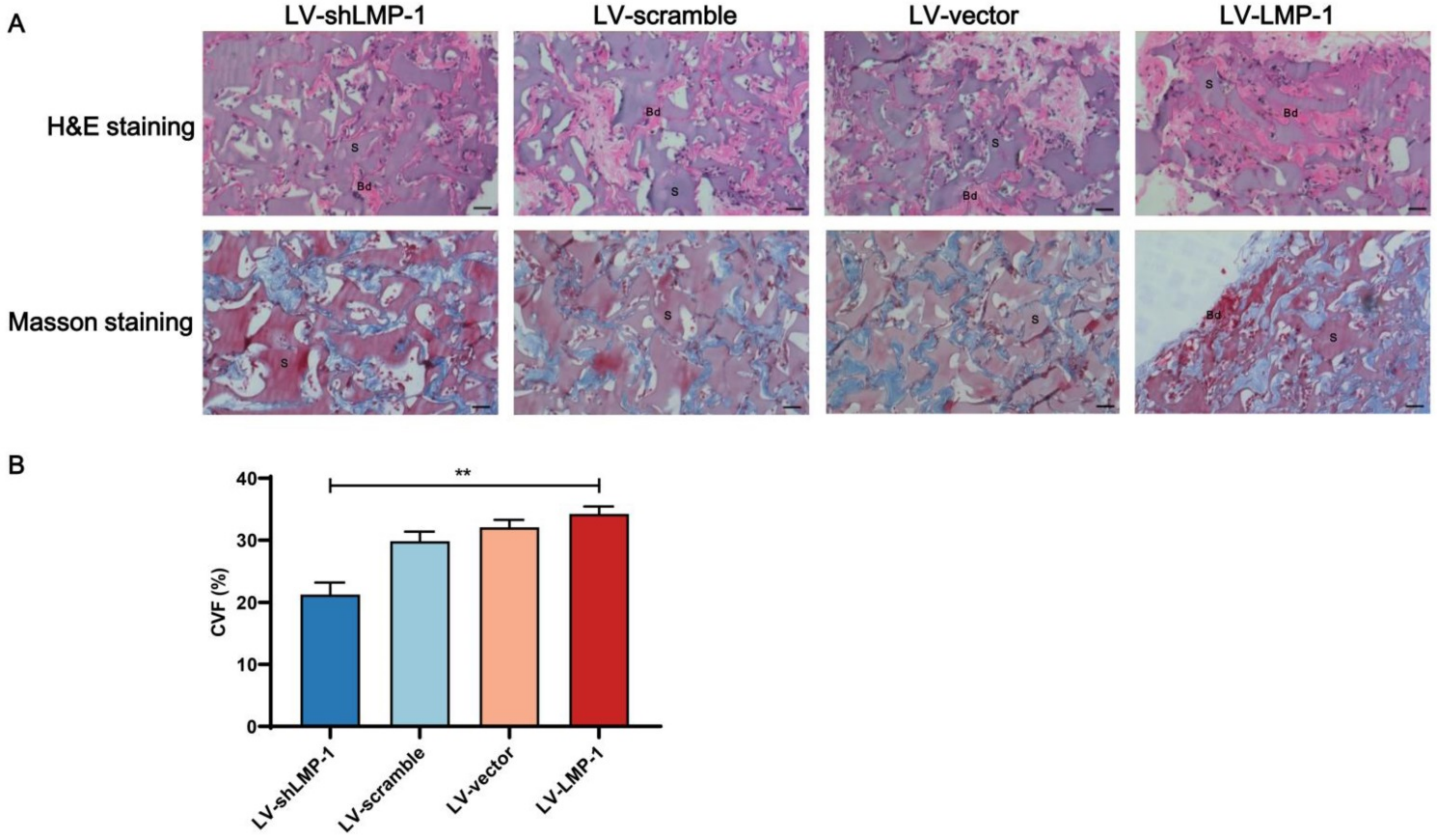
** Histological analysis of LMP-1 on the odonto/osteogenesis of LMP-1 *in vivo*. (A)** H&E staining and Masson staining in LV-scramble group, LV-shLMP-1 group, LV-vector group, and LV-LMP-1 group after 8 weeks growth. **(B)** CVF of LV-scramble group, LV-shLMP-1 group, LV-vector group, and LV-LMP-1 group after 8 weeks growth (**P < 0.01). Bd Bone/dentin-like tissue, S Surrounding scaffold. Scale Bar = 50 µm.

**Figure 6:**
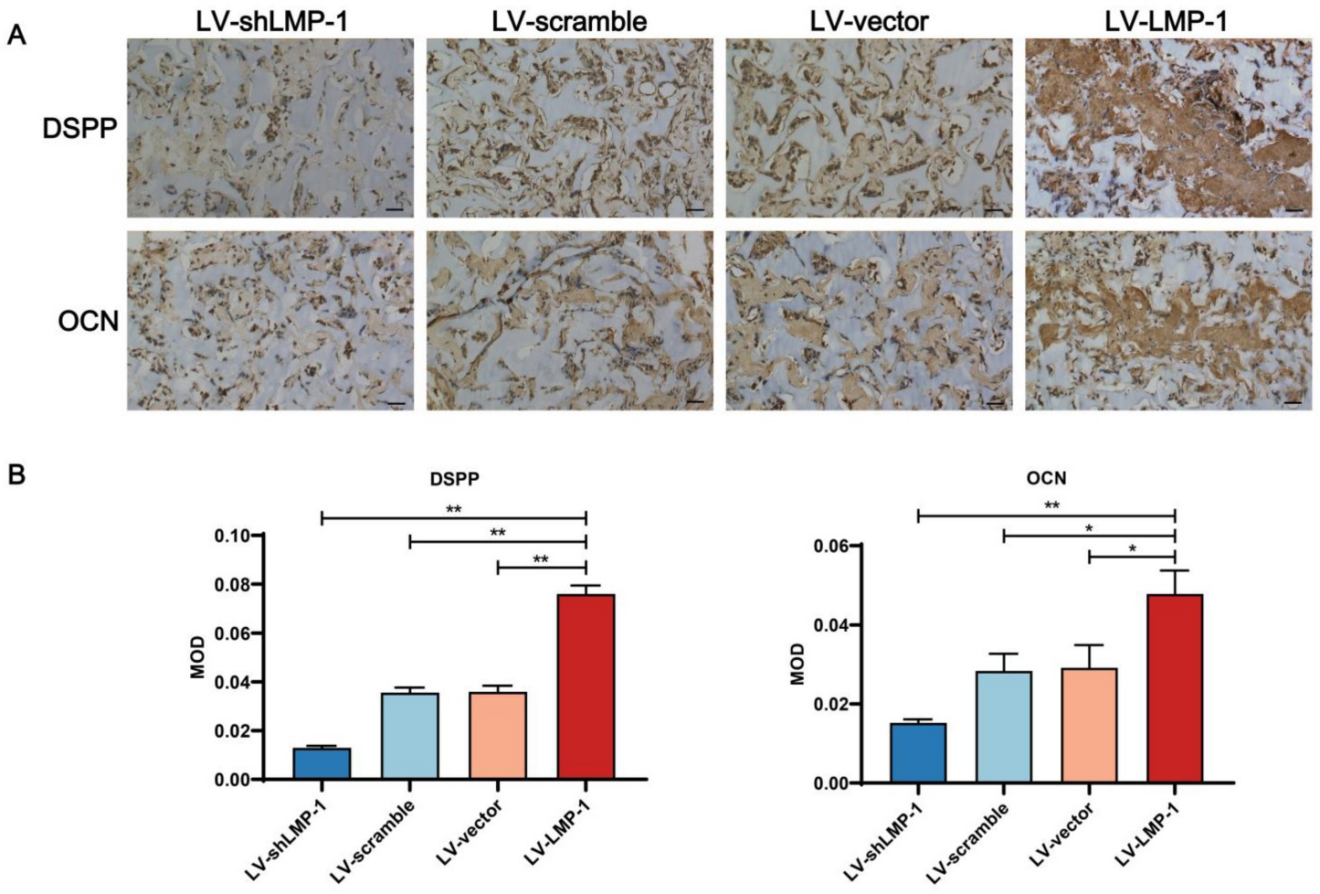
** Immunohistochemical observation of LMP-1 on the odonto/osteogenesis of LMP-1 *in vivo*. (A)** Immunohistochemical staining of DSPP and OCN in LV-scramble group, LV-shLMP-1 group, LV-vector group, and LV-LMP-1 group after 8 weeks growth. **(B)** MOD of DSPP and OCN in LV-scramble group, LV-shLMP-1 group, LV-vector group, and LV-LMP-1 group after 8 weeks growth (*P < 0.05, **P < 0.01). Scale Bar = 50 µm.

To confirm the biological reaction of the implants, the tissue samples with H&E and Masson's trichrome staining after an 8-week growth period in the subcutaneous space of BALB/c homozygous nude mice are shown in **Figure 5**. The ability of proliferation and differentiation in the LV-LMP-1 group of transfected cells was enhanced, and the matrix or bone/dentin-like tissue could be observed. However, there was a smaller amount of matrix or bone/dentin-like tissue in the LV-shLMP-1 group than in the LV-LMP-1 group. Meanwhile, in the histological sections stained using Masson's trichrome, the LV-LMP-1 group had a more abundant blue-stained collagen deposit in comparison with the LV-shLMP-1 group. The CVF was 34.26% ± 1.19% in the LV-LMP-1 group and 21.73% ± 1.94% in the LV-shLMP-1 group, and the difference was statistically significant (*P* < 0.01). In addition, the red-stained matrix or bone/dentin-like tissue could be observed in the LV-LMP-1 group. Moreover, the immunohistochemical staining showed that the odonto/osteogenic differentiation-related proteins DSPP and OCN of all groups were expressed in varying degrees **(Figure 6)**. The abundance of both DSPP and OCN was increased in the LV-LMP-1 group compared with the other three groups, and the MOD of DSPP and OCN in the LV-LMP-1 group was also considerably higher than that in the other three groups. Consistent with the *in vitro* results, LMP-1 overexpression also had the ability to promote DPSCs to form bone/dentin-like tissue *in vivo*.

### LMP-1 activated ERK1/2, p38 MAPK pathways and BMP-2/Smad1/5 signaling of DPSCs

**Figure 7:**
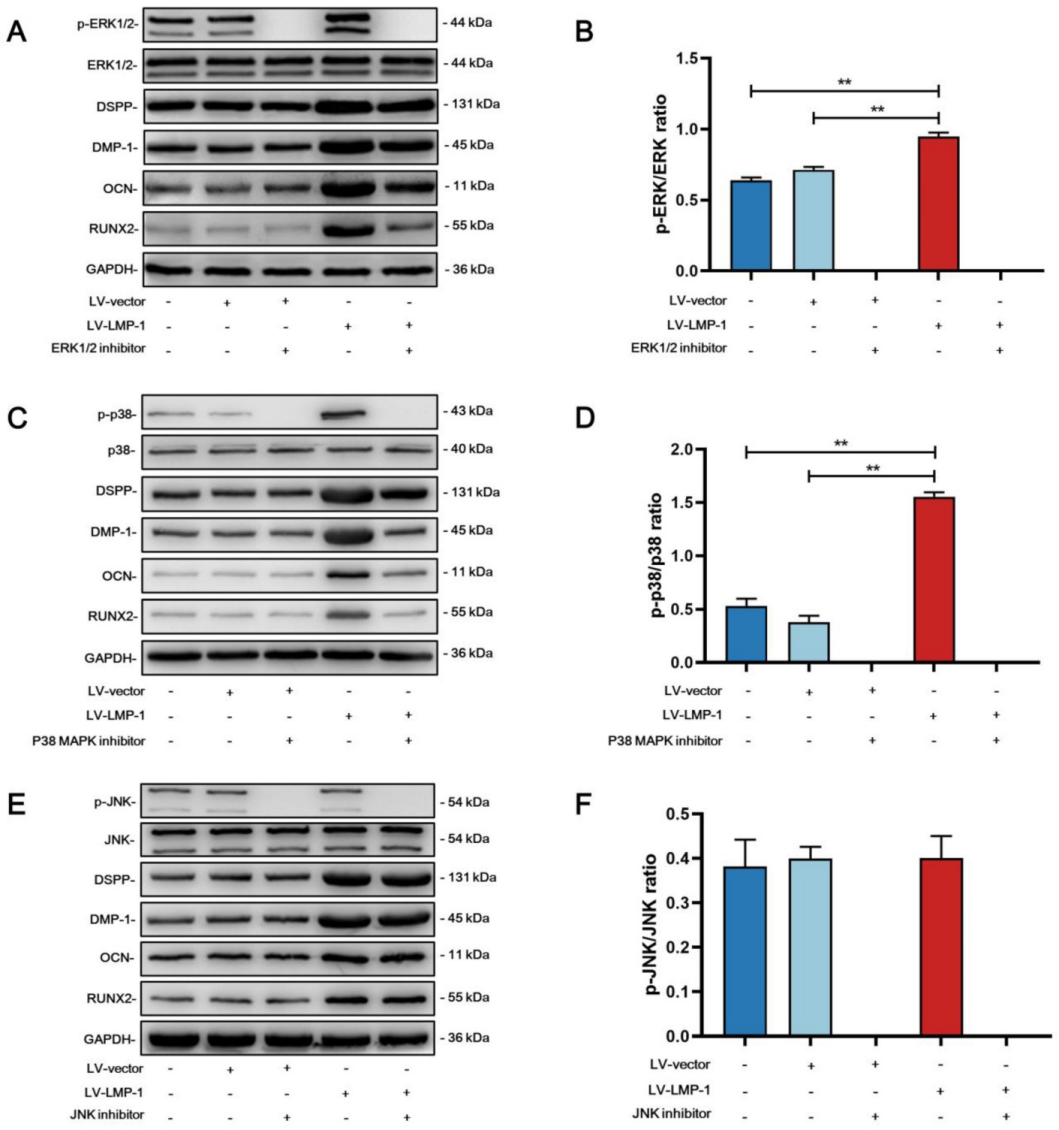
** Effects of LMP-1 on regulating the odonto/osteogenic differentiation of DPSCs through the MAPK pathway.** Western blot analysis of p-ERK1/2 **(A),** p-p38 MAPK **(C),** p-JNK **(E),** and protein levels of the odonto/osteogenic markers (DSPP, DMP-1, OCN, and RUNX2) **(A, C, E)** in DPSCs after being treated with ERK1/2 pathway inhibitor U0126 (20 µM), p38 MAPK pathway inhibitor SB203580 (10 µM), JNK pathway inhibitor SP600125 (25 µM), respectively. GAPDH served as an internal control. Quantitative analysis for the ratios of p-ERK/ERK (B), p-p38/p38 (D) and p-JNK/JNK (F) (**P < 0.01).

**Figure 8:**
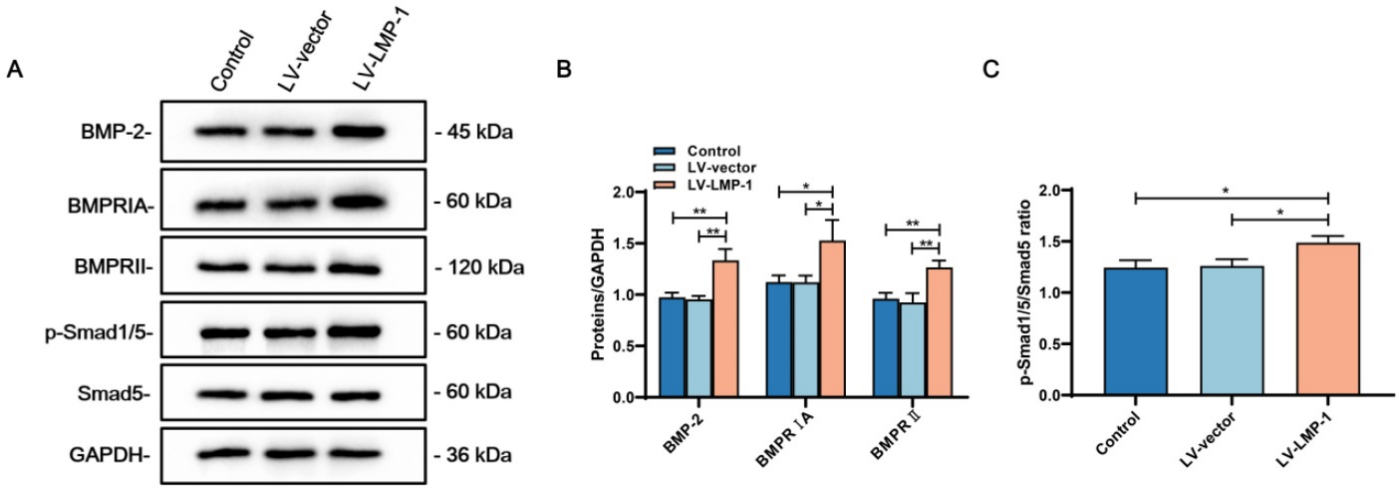
** The expression of BMP signaling pathway components of DPSCs with LMP-1 induction. (A)** Western blot analysis of BMP-2, BMPRIA, BMPRII, p-Smad1/5, and Smad5 in DPSCs after LMP-1 induced, respectively. GAPDH served as an internal control. **(B)** Quantitative analysis for the BMP-2, BMPRIA and BMPRII (*P < 0.05, **P < 0.01). (C) Quantitative analysis for the ratios of p-Smad1/5/Smad5 (*P < 0.05).

To investigate the molecular mechanism of whether LMP-1 could activate the MAPK pathways, we applied specific inhibitors to DPSCs during odonto/osteogenic differentiation with or without LMP-1 induction. Although the inhibitors attenuated the phosphorylation of ERK1/2, p38, and JNK, there were no remarkable differences in the protein levels of DSPP, DMP-1, OCN, and RUNX2. The protein levels of DSPP, DMP-1, OCN, and RUNX2 were upregulated by LMP-1 induction. The ERK1/2 pathway inhibitor U0126 **(Figure 7A)** and p38 pathway inhibitor SB203580 **(Figure 7C)** further attenuated the upregulated protein levels of DSPP, DMP-1, OCN, and RUNX2. However, the JNK pathway inhibitor SP600125 showed no effect on the LMP-1 induced increase in the levels of DSPP, DMP-1, OCN, and RUNX2 **(Figure 7E)**. There were statistically significant differences in the ratio of p-ERK/ERK **(Figure 7B)** and p-p38/p38 **(Figure 7D)** between LV-LMP-1 without inhibitor induction and LV-vector without inhibitor induction, but there was no difference in p-JNK/JNK **(Figure 7F)**. To further understand BMP-2 mediated signaling in DPSCs with LMP-1 induction, we determined the expression of BMP signaling pathway components. The LV-LMP-1 group expressed the BMP receptors BMPRIA and BMPRII markedly higher than the other groups **(Figure 8A-B)**. Meanwhile, the BMP-2 protein level was higher in the LV-LMP-1 group than that in the two groups **(Figure 8A-B)**, and the ration of p-Smad1/5/Smad5 had statistically significant differences between LV-LMP-1 group and the other two groups **(Figure 8C)**. It is suggested that the ERK1/2, p38 MAPK pathways and BMP-2/Smad1/5 signaling are involved in the process of LMP-1 induced odonto/osteogenic differentiation of DPCSs.

## Discussion

Odonto/osteogenic regeneration is a complex process depending on cell proliferation and the regulation of multiple osteogenic factors. LMP-1 is an intracellular protein that can potentiate the expression of various osteogenic-related factors in various cells, such as BMP-2, BMP-4, and BMP-6 16, 26, 27. After LMP-1 knockout, the density of spinal trabecular bone reportedly decreased and the responsiveness of BMPs reduced, indicating that LMP-1 played a positive role in osteogenic regulation 17. Similar to the osteogenic mechanism, LMP-1 may promote the formation of dentin by indirectly enhancing the responsiveness of BMPs, thereby enabling the mineralization of DPSCs. Therefore, we hypothesized that LMP-1 could activate BMPs related signaling pathways in DPSCs and further regulate the odonto/osteogenic differentiation. To test this hypothesis, we examined the expression of BMP signaling pathway components after LMP-1 overexpression, it is suggested that LMP-1 could activate BMP-2/Smad1/5 signaling pathway. In addition to BMPs, many other odonto/osteogenic markers are involved in osteogenesis, such as ALP, DSPP, DMP-1, OCN, and RUNX2. ALP is an extracellular enzyme that plays a vital role in the formation of the extracellular matrix and mineralized tissue 28, degradation of inorganic phosphates, and promotion of cell mineralization 29. DSPP, as an essential element of normal dental development, plays a crucial role in the formation and mineralization of dentin and usually a specific tooth biomarker 30. DMP-1 is an essential component of non-collagenous protein in dentin, which is expressed during tooth development 31. OCN is found in bone and dentin during the late phase of osteogenic differentiation, which is considered the terminal phase in hard tissue regeneration 32. RUNX2, as the master regulator of bone development 33, is a major transcription factor associated with osteogenic differentiation 34.

Previous studies reported that the downregulation of LMP-1 could reduce the expression of ALP, RUNX2, and OCN, and the formation of mineralized nodules 14, 18. Additionally, DNA synthesis was inhibited and mineralization ability decreased in the periodontal ligament progenitor cells after LMP-1 knockdown 35. In the current study, LMP-1 did not affect the proliferation ability of DPSCs. However, we suggest that the downregulation of LMP-1 expression resulted in a decrease in the formation of mineralized nodules, ALP activity, and odonto/osteogenic markers (DSPP, DMP-1, OCN, and RUNX2) expressions in DPSCs. Our results also indicated that the overexpression of LMP-1 upregulated the construction of mineralized nodules and ALP activity of DPSCs, as well as the expression of DSPP, DMP-1, OCN, and RUNX2, which demonstrated that the overexpression of LMP-1 could stimulate osteogenic differentiation and the formation of mineralized nodules, and sustained overexpression formed bone/dentin tissue in the animal experiments. Moreover, the ability of odonto/osteogenic differentiation was also strengthened by LMP-1 overexpression *in vivo*. However, in addition to BMPs signaling pathways, the other mechanisms of LMP-1 on committed differentiation of DPSCs remains unclear.

MAPK is a type of Ser/Thr protein kinase that exists in most cells and transduces extracellular signaling molecules into the nucleus. It plays a vital regulatory role in cell proliferation, differentiation, and other activities. MAPK mainly includes ERK, p38, and C-JNK signaling pathways 36. There are five subtypes of ERKs, with ERK1/2 being the most studied. ERK1/2 participates in the signal transduction of various growth factors, mitogens, and hormone receptors, which means that ERK1/2 has a crucial regulatory role in cell proliferation, growth, and differentiation 37. The ERK1/2 signaling pathway could also activate and phosphorylate the bone-specific transcription factor Cbfa1, which further promoted the expression of osteogenic genes 38. Simultaneously, RUNX2 is one of the downstream genes that can be regulated by the ERK1/2 signaling pathway 39, 40. Relevant studies have also demonstrated that ERK1/2 has a positive effect on the odonto/osteogenic differentiation of MSCs 41-43. However, some studies have shown that the osteogenic activity of cells was enhanced after inhibiting the activity of ERK1/2 44, 45. This inconsistency may be due to the complexity and uncertainty of this pathway and unknown mechanisms of gene regulation in odonto/osteogenic regeneration. Another classic pathway in the MAPK family, the p38 MAPK signaling pathway participates in the signal transduction of various stress responses and inflammatory factors. During the development from predentin to secondary dentin, regulation of the p38 MAPK pathway is essential in the secretion activity of odontoblasts 46, 47. It was also reported that the expressions of ALP and OCN were significantly reduced after inhibiting the p38 MAPK signaling pathway 48.

The MAPK pathways are widely explored due to its important role in committed differentiation of MSCs. Pan et al. found that LMP-1 could positively regulate BMP-2 expression and BMP-2-mediated osteogenesis through the activation of the ERK1/2 pathway and subsequent upregulation of Runx2 transactivity in the pre-osteoblasts 18. However, Liu et al. reported that LMP-1 did not affect the LPS-induced phosphorylation of extracellular signal-regulated ERK1/2 in the pre-osteoclasts, whereas it attenuated the phosphorylation of JNK while improving the phosphorylation of p38 MAPK 49. The different results of these two studies may be a result of the different cell lines and microenvironments. Therefore, we hypothesized that LMP-1 could regulate the committed differentiation of DPSCs by activating MAPK pathways. To test this hypothesis, the effect of the MAPK pathways in the odonto/osteogenic differentiation of DPSCs was investigated using specific pathway inhibitors. Our results showed that LMP-1-overexpression upregulated the phosphorylation of ERK1/2 and p38 MAPK in DPSCs, but not JNK. Moreover, the upregulation of DSPP, DMP-1, OCN, and RUNX2 induced by LMP-1 overexpression was attenuated by inhibiting the ERK1/2 and p38 MAPK signaling pathways with specific inhibitors, providing further evidence that the ERK1/2 and p38 MAPK signaling pathways may be involved in the odonto/osteogenic differentiation of DPSCs caused by LMP-1 overexpression.

Although the present study showed that LMP-1 could enhance the odonto/osteogenic differentiation of DPSCs and form a matrix of bone/dentin-like tissue *in vivo*, there were differences in the microenvironments between bone/dentin tissue and subcutaneous tissue. An *in situ* study of odonto/osteogenic tissue formation in dentin/bone defect repair should be conducted.

## Conclusion

LMP-1 may promote the odonto/osteogenic differentiation of DPSCs through the activation of the ERK1/2, p38 MAPK and BMP-2/Smad1/5 signaling pathways, which offers valuable insight and therapeutic strategies for regenerative medicine.

## Abbreviations

ALP: alkaline phosphatase
α-MEM: alpha minimum essential medium
BMMSCs: bone marrow mesenchymal stem cells
BMPs: bone morphogenetic proteins
CCK-8: cell counting kit-8
CPC: cetylpyridinium chloride
CVF: collagen volume fraction
DMP-1: dentin matrix protein 1
DPSCs: dental pulp stem cells
DSPP: dentin sialophosphoprotein
ERK: extracellular signal-regulated kinase
FBS: fetal bovine serum
GAPDH: Glyceraldehyde-3-phosphate dehydrogenase
H&E: hematoxylin-eosin
JNK: c-Jun N-terminal kinase
LMP-1: LIM mineralization proteins 1
MAPK: mitogen-activated protein kinase
MOD: mean optical density
MSCs: mesenchymal stem cells
OCN: osteocalcin
OD: optical density
PBS: phosphate-buffered saline
qRT-PCR: quantitative real-time polymerase chain reaction
RUNX2: runt-related transcription factor 2
TGF-β: transforming growth factor β
